# Effects of Supplementation with Chlorogenic Acid-Rich Extract from *Eucommia ulmoides* Oliver During Peri-Implantation on the Reproductive Performance and Gut Microbiota of Sows

**DOI:** 10.3390/vetsci12090857

**Published:** 2025-09-04

**Authors:** Yan Zhang, Hexuan Qu, Hongda Pan, Dao Xiang, Seongho Choi, Shuang Liang

**Affiliations:** 1 College of Animal Science and Technology, Jilin Agricultural Science and Technology College, Jilin 132109, China; zhangyan888001@jlnku.edu.cn; 2 Department of Animal Science, College of Animal Sciences, Jilin University, Changchun 130062, China; quhx21@mails.jlu.edu.cn (H.Q.); panhd23@mails.jlu.edu.cn (H.P.); xiangdao22@mails.jlu.edu.cn (D.X.); 3 Department of Animal Science, Chungbuk National University, Cheongju 28644, Republic of Korea

**Keywords:** chlorogenic acid, oxidative stress, reproductive performance, gut microbiota, sows

## Abstract

Chlorogenic acid (CGA)-rich extracts from *Eucommia ulmoides* Oliver (CAE) are known for their gut health and antioxidant benefits in livestock. This study examines the effects of CAE supplementation during the peri-implantation period on sow reproductive performance and the gut microbiota. Sixty Dongliao black sows were randomized to receive either no supplementation (control) or CAE at 600 or 2000 mg/kg daily from gestation day −5 through day 15. High-dose CAE intake significantly increased total antioxidant capacity (T-AOC), superoxide dismutase (SOD), catalase (CAT), immunoglobulin A (IgA), and immunoglobulin M (IgM) levels in sow serum but decreased malondialdehyde (MDA) levels. Fecal short-chain fatty acids (SCFAs) also increase significantly. These changes correlate with improved reproductive performance, including a larger litter size, higher numbers of live-born piglets, a greater individual birth weight of live-born piglets, a higher total litter birth weight of live-born piglets, and a lower mortality rate. 16S rRNA sequencing of the fecal microbiota revealed that CAE markedly altered microbial diversity and composition, reducing the abundance of potentially harmful bacteria but increasing the abundance of beneficial bacteria. In conclusion, supplementation with CAE during the peri-implantation phase can reduce oxidative stress, alter the gut microbiota composition, and improve sow reproductive performance, thus potentially increasing breeding farm profitability.

## Simple Summary

Pregnant sows produce high levels of reactive oxygen species (ROS) because of their vigorous metabolism. Excessive ROS can cause oxidative stress, impeding embryo development and reducing piglet survival rates. Early gestation, especially the pre-implantation phase, is a critical period for embryonic loss and affects embryo quality and sow productivity. Reducing oxidative stress during early pregnancy is vital for improving sow performance. In addition, reproductive performance in sows is influenced by factors such as the environment and nutrition. Through its metabolic products, the gut microbiota interacts with the host to affect reproductive processes, including follicular maturation, fertilization, implantation, and embryo migration. These microbial signals can alter reproductive outcomes by influencing nutrition, energy balance, and immune homeostasis. *Eucommia ulmoides* Oliver, a traditional Chinese medicinal plant rich in bioactive compounds, such as chlorogenic acid (CGA), has antioxidant, anti-apoptosis, and anti-inflammatory properties. While a CGA-rich extract from *Eucommia ulmoides* Oliver (CAE) shows potential as a novel feed additive, its effects on sow reproductive performance and the gut microbiota during the peri-implantation period remain unclear. This study seeks to address this knowledge gap and to provide insights into improving sow reproductive performance through dietary interventions.

## 1. Introduction

In the pig industry, the reproductive performance of sows, particularly their litter size, is a crucial factor in determining the economic efficiency of pig farms. Throughout pregnancy, the increase in metabolic processes in sows results in increased production of reactive oxygen species (ROS). If these ROS are not sufficiently mitigated, oxidative stress ensues, adversely affecting sow health and reproductive capabilities by decreasing fertility and increasing the risk of intrauterine growth restriction (IUGR) in offspring [1]. Elevated oxidative stress during early gestation can delay the migration of fertilized eggs and mitosis before implantation, reducing the infiltration of fertilized egg morula into the uterine wall and postponing embryo implantation [2]. The peak incidence of embryonic mortality in sows is typically observed between gestational days 12 and 15, which corresponds to the peri-implantation stage [3]. Therefore, the early gestation period, especially the pre-implantation phase, is a critical and sensitive stage for embryonic loss and may ultimately affect the size of sow litter. Moreover, the inflammatory status of sows is closely linked to pregnancy complications. Increased oxidative stress can damage the immune system of sows, triggering inflammation. Most cases of unexplained embryonic loss during pregnancy are categorized as immunomodulatory disorders [4]. Therefore, during the peri-implantation phase, it is essential to mitigate oxidative stress and enhance immune function to support early embryonic development and maintain high fertility in sows.

The gut microbiota is intricately linked to the physiological activities of the host and is involved in numerous metabolic processes. It has been characterized as an independent physiological organ that plays a crucial role in nutrient metabolism and immune development [5]. Additionally, the gut microbiota influences all stages of the female reproductive cycle and modulates host reproductive performance [6]. Consequently, changes in the gut microbiota composition can directly affect the physiological functions of sows. *Eucommia ulmoides* Oliver (*Eucommiaceae*), commonly known as *Du-Zhong*, is a traditional Chinese medicinal plant that is abundant in resources and rich in bioactive compounds such as chlorogenic acid (CGA). Studies have demonstrated that CGA has antioxidant, anti-apoptosis, and anti-inflammatory properties [7,8,9]. Hence, the CGA-rich extract from *Eucommia ulmoides* Oliver (CAE) has potential as a novel feed additive. However, the effects of supplementation with CAE before embryo implantation on sow reproductive performance and the gut microbiota remain incompletely understood. Therefore, we hypothesize that supplementing sows with CAE during the peri-implantation period may help alleviate oxidative stress, promote the development of the immune system, and optimize the gut microbiota composition, thus potentially enhancing the reproductive performance of sows. In the present study, Dongliao black sows were utilized to achieve two primary objectives: first, to assess the effects of CAE on the reproductive performance, oxidative stress-related factors, and immune indices of sows; and second, to elucidate how CAE influences the gut microbiota of sows. Clarifying these relationships helps to improve sow reproductive performance through feed interventions and is highly important for enhancing the profitability of pig farms.

## 2. Materials and Methods

### 2.1. Experimental Animals and Design

**Figure 1:**
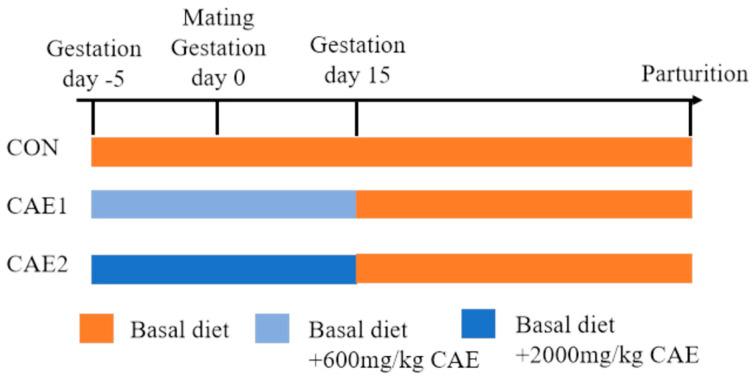
Design of the trial. The term CON refers to the sows that were fed a standard basal diet without supplementation, whereas CAE1 and CAE2 denote the groups that were fed the basal diet supplemented with CAE at two different doses, 600 mg/kg and 2000 mg/kg, respectively.

The experiments were conducted at the Jilin University Agricultural Experiment Base, which offers a regulated environment for livestock and is equipped with facilities that adhere to standard agricultural practices and prioritize animal welfare. Sixty healthy Dongliao black sows with similar body weights (220 ± 5 kg), parities (2–3 parities), and prior reproductive performance (litter size of 10–12 piglets) were randomly divided into three groups five days prior to mating (gestation day −5): a control group (CON), a group treated with 600 mg/kg of CAE (CAE1), and a group treated with 2000 mg/kg of CAE (CAE2). Each group included 20 sows (*n* = 20), which were randomly distributed across four pens with five sows per pen (4 × 5 = 20). Throughout gestation, sows were housed in these pens. One week before expected parturition, all sows were moved to individual farrowing crates within the same facility to ensure consistent farrowing management. From gestation day −5 to day 15, sows were fed a basal diet supplemented with the respective CAE concentrations daily. From gestation day 16 until parturition, sows were fed the basal diet without CAE. The detailed composition of the basal diet is provided in Table 1, and the experimental design is illustrated in Figure 1. During the trial period, sows were fed a controlled diet twice daily at 6:00 and 17:00, and they had unrestricted access to water for drinking. The amount of the basal diet was adjusted according to the gestation stage: 1.8–2.2 kg/day during gestation days −5 to 30, 2.5–2.8 kg/day during days 31–85, 3.0–3.2 kg/day during days 86–111, and 2.0 kg/day from day 112 until parturition. Adjustments were made on the basis of the physical condition of the sows, with slight decreases for those that were overweight and slight increases for those that were underweight. In this study, the CAE (with a CGA content of 50%) was supplied by Changsha Shanghe Biotechnology Co., Ltd. (STACHA-221015001, Changsha, China). Immunization and deworming procedures, along with other management activities, were carried out in accordance with the regular management system of the experimental base. Daily records included the ambient temperature and humidity within the pigsty, as well as the health status of the sows.

### 2.2. Sample Collection

Before the start of the CAE feeding trial (gestation day −5) and after the trial ended (day 15 of gestation), eight sows were randomly selected from each group for fecal sample collection (two randomly chosen from each of the four pens). The samples were placed into 5 mL sterile polypropylene tubes and stored at −80 °C for subsequent analysis. In addition, blood samples were collected through jugular vein puncture on the 15th day of gestation. The process took less than 2 min per sow, with two to three sows being randomly chosen from each of the four pens in each group. After collection, the blood samples were centrifuged at 4500× *g* for 10 min, after which the collected serum was preserved at −80 °C for subsequent analysis.

### 2.3. Reproductive Performance Analysis

Postpartum, the following parameters were documented for each sow: litter size (head), total number of live-born piglets (head), individual birth weight of live-born piglets (g), and total litter birth weight of live-born piglets (g). These data were used to calculate the mortality rate (%) and the within-litter birth weight coefficient of variation (CV) for live-born piglets (%). The mortality rate (%) was calculated as follows: (1 − total number of live-born piglets/litter size) × 100%. The within-litter birth weight CV for live-born piglets (%) was calculated as follows: (standard deviation of live-born piglet birth weight) × 100%/mean live-born piglet birth weight.

### 2.4. Serum Biochemical Parameters Analysis

Serum biochemical parameters, including malondialdehyde (MDA), catalase (CAT), total antioxidation capacity (T-AOC), superoxide dismutase (SOD), immunoglobulin A (IgA), immunoglobulin G (IgG), and immunoglobulin M (IgM), were measured using MDA (A003-1), CAT (A007-1-1), T-AOC (A015-1), SOD (A001-3), IgA (H108-1-2), IgG (H106-1-1), and IgM (H107-1-1) ELISA kits (Nanjing Jiancheng Bioengineering Research Institute Co., Ltd., Nanjing, China).

### 2.5. DNA Extraction and Microbiota Analysis

Extraction of total microbial DNA from sow fecal samples was carried out with a DNeasy PowerSoil Kit (47016; QIAGEN Business Management Co., Ltd., Shanghai, China) following the protocol provided by the manufacturer. First, the fecal samples were added to the bead tubes provided in the kit, followed by the addition of lysis buffer and mechanical homogenization to ensure complete cell lysis. The inhibitor removal technology of the kit was subsequently employed to eliminate PCR inhibitors from the samples. Finally, the DNA was purified through silica membrane spin columns and eluted using elution buffer. The quantity and purity of the extracted DNA were assessed using a Nanodrop 1000 spectrophotometer (Nanodrop Technologies, Wilmington, DE, USA).

The V3 and V4 regions of the 16S rRNA gene were amplified via PCR using the primers 341F (5′-CCTAYGGGRBGCASCAG-3′) and 806R (5′-GGACTACNNGGGTATCTAAT-3′). The PCR amplification conditions were as follows: initial denaturation at 98 °C for 1 min, 30 cycles of denaturation at 98 °C for 10 s, annealing at 50 °C for 30 s, elongation at 72 °C for 30 s, and a final extension at 72 °C for 5 min. The NEBNext^®^ Ultra™ II DNA Library Prep Kit for Illumina^®^ (E7645L; New England Biolabs, Ipswich, MA, USA) was used to construct libraries from the PCR products according to the manufacturer’s instructions. The libraries were subsequently sequenced on the Illumina MiSeq platform (Illumina, San Diego, CA, USA) to produce 250 bp paired-end raw sequences. The raw sequencing data, containing primer sequences and low-quality reads, were processed using the QIIME2 software (version 2019.4) [10] with the DADA2 plugin to filter out contaminants and generate high-quality sequences, referred to as “clean reads” [11]. These clean reads were then merged into longer contiguous sequences using FLASH. Finally, chimeric sequences and singletons were removed to generate amplicon sequence variants (ASV) [11]. The ASV were taxonomically classified using the Greengenes2 database as a reference [12].

Microbial alpha diversity was assessed on the basis of rarefied ASV using the Chao1, Shannon, and Pielou_e indices via QIIME2 (version 2019.4). Beta diversity analysis of microbial communities was performed using the Bray—Curtis distance, and the results were visualized through principal coordinate analysis (PCoA) plots and cluster analysis. Dissimilarities in fecal microbial community composition were analyzed using permutational multivariate analysis of variance (PERMANOVA) based on Bray—Curtis distances, with 999 permutations performed using the “vegan” package in R (v3.0.3).

### 2.6. Short-Chain Fatty Acid (SCFA) Analysis

The analysis of SCFAs was conducted using methods adapted from previous studies, with necessary modifications [13,14]. Approximately 0.2 g of sow fecal sample was weighed into each 2 mL centrifuge tube. Distilled water (0.5 mL) was added to each tube, and the mixture was swirled for 30 s. The samples were then centrifuged at 12,000× *g* for 10 min. Next, 0.2 mL of the supernatant was transferred to a new 2 mL centrifuge tube and mixed with 0.1 mL of 15% metaphosphoric acid (7664-38-2; Sinopharm, Beijing, China), 20 μL of a 375 μg/mL 4-methylvaleric acid solution (646-07-1; Sigma, St. Louis, MO, USA) as an internal standard, and 280 μL of ether (60-29-7; Greagent, Shanghai, China). The mixture was placed at 4 °C for 30 min, followed by centrifugation at 12,000× *g* for 10 min. Finally, 1 μL of the supernatant was injected into a gas chromatograph (GC) for SCFA analysis.

Chromatography was performed on a Trace 1310 GC (TRACE1300, Thermo Fisher Scientific, Waltham, MA, USA) equipped with an Agilent HP-INNOWAX column (with a 30 m × 0.25 mm ID × 0.25 μm film). The GC conditions were as follows. The initial column temperature was set at 100 °C, held at that temperature for 2 min, then increased to 200 °C at a rate of 15 °C/min, and held at that temperature for an additional 5 min. The flame ionization detector (FID) temperature was maintained at 260 °C. The flow rates of hydrogen, air, and nitrogen were set to 35, 350, and 25 mL/min, respectively. The sample was injected at 260 °C with a split ratio of approximately 25:1, and nitrogen was used as the carrier gas. Each analysis had a total run time of 12.95 min.

### 2.7. Statistical Analysis

Statistical analyses and result visualization were performed using the GraphPad Prism software, version 8 for Windows. Normality was assessed using the Shapiro—Wilk test and the Kolmogorov—Smirnov test. To compare reproductive performance metrics among the three groups, one-way ANOVA or the Kruskal—Wallis test was used. For comparisons between two groups, either Student’s t test or the Mann—Whitney U test was employed for serum biochemical parameters, SCFA levels, and fecal microbiota composition. The data are expressed as the means ± SEMs. The associations between altered microbiota and antioxidant indices, as well as between altered microbiota and reproductive performance indices, were analyzed using Spearman correlation coefficients to assess significance. A *p* value of less than 0.05 was considered to indicate statistical significance.

## 3. Results

### 3.1. Reproductive Performance

**Figure 2:**
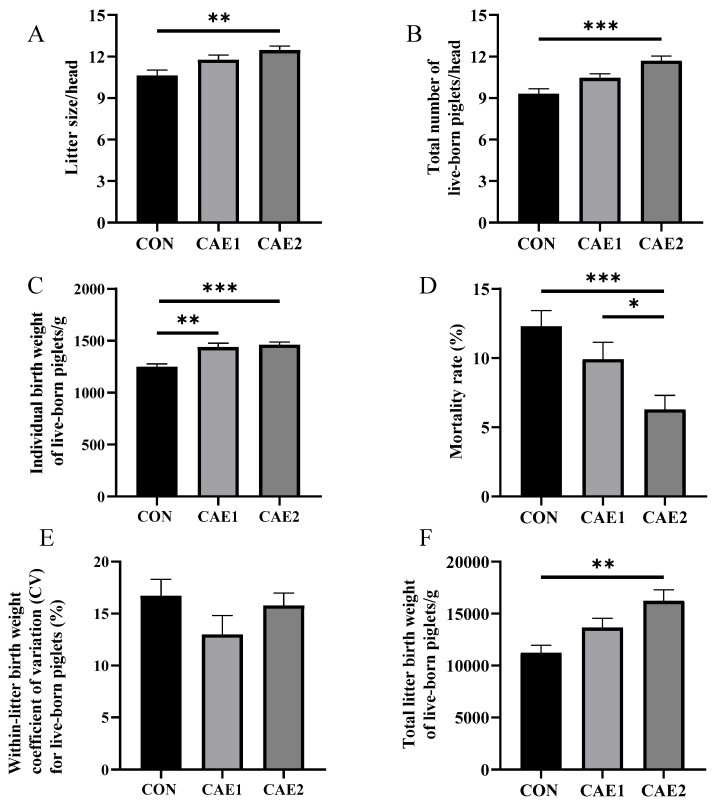
Effects of CAE supplementation during the peri-implantation phase on sow reproductive performance. (**A**) Litter size, (**B**) total number of live-born piglets, (**C**) individual birth weight of live-born piglets, (**D**) mortality rate, (**E**) within-litter birth weight CV for live-born piglets, and (**F**) total litter birth weight of live-born piglets (*n* = 20). The term CON refers to the sows that were fed a standard basal diet without supplementation, whereas CAE1 and CAE2 denote the groups that were fed the basal diet supplemented with CAE at two different doses, 600 mg/kg and 2000 mg/kg, respectively. * *p* < 0.05; ** *p* < 0.01; *** *p* < 0.001.

The results are presented in Figure 2. Compared with those in the CON group, the individual birth weight of live-born piglets in the CAE1 and CAE2 groups were significantly greater (*p* = 0.002, *p* < 0.001). Furthermore, compared with the CON group, the CAE2 group had a significantly larger litter size (*p* = 0.005), greater total number of live-born piglets (*p* < 0.001), and higher total litter birth weight of live-born piglets (*p* = 0.002). The mortality rate was significantly lower in the CAE2 group than in the CON group (*p* < 0.001) and the CAE1 group (*p* = 0.041). Moreover, no significant differences were found in the litter size (*p* = 0.188), total number of live-born piglets (*p* = 0.232), total litter birth weight of live-born piglets (*p* = 0.157), or mortality rate (*p* = 0.381) between the CON group and the CAE1 group. Additionally, no significant differences were observed in the within-litter birth weight CV for live-born piglets among the CAE1, CAE2, and CON groups (*p* > 0.228). These results suggest that supplementation with CAE can enhance the reproductive performance of sows. Specifically, the optimal dose of CAE for sows appears to be 2000 mg/kg per day during gestation days −5 to 15, as evidenced by the superior performance of the CAE2 group. Consequently, in subsequent analyses, only samples from the CAE2 group were selected for further testing, and this group was renamed the CAE group.

### 3.2. Serum Antioxidant and Immune Indices

**Figure 3:**
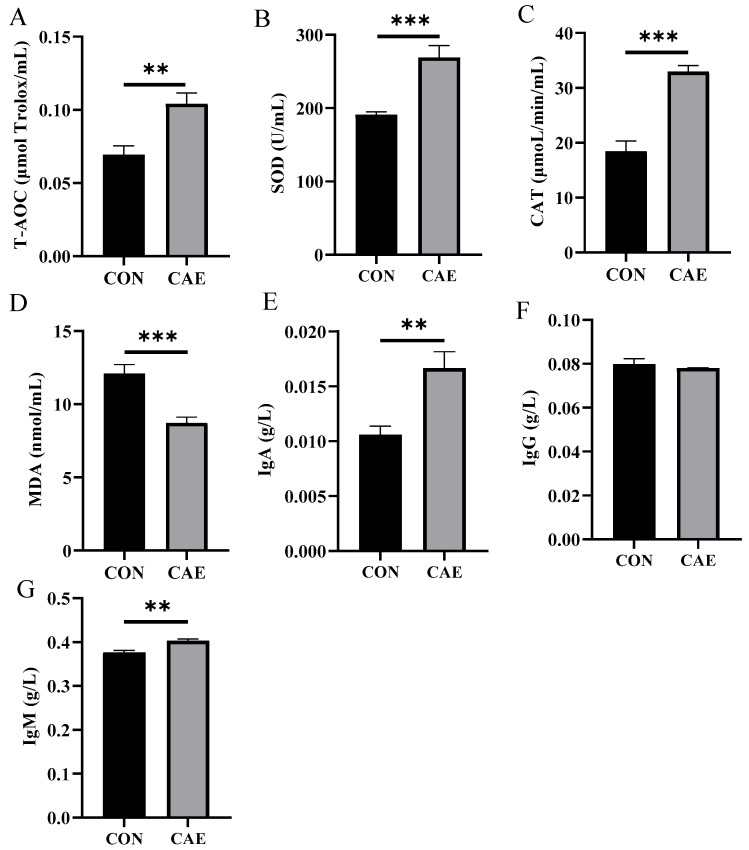
Effects of CAE supplementation during the peri-implantation phase on sow serum antioxidant and immune indices. Levels of (**A**) T-AOC, (**B**) SOD, (**C**) CAT, (**D**) MDA, (**E**) IgA, (**F**) IgG, and (**G**) IgM (*n* = 8–12). The term CON refers to the sows that were fed a standard basal diet without any supplementation, whereas CAE denotes the groups that were fed the basal diet supplemented with CAE at doses of 2000 mg/kg. ** *p* < 0.01; *** *p* < 0.001.

In this investigation, CAE supplementation markedly elevated the T-AOC (*p* = 0.002), SOD (*p* < 0.001), and CAT (*p* < 0.001) levels and concurrently reduced the MDA level (*p* < 0.001) in sow serum (Figure 3A–D). These outcomes underscore that CAE effectively increases the antioxidant defenses of sows during the peri-implantation phase. The inflammatory status of sows is intricately linked to pregnancy complications. Maternal immunity can be conveyed to the fetus via the placenta and milk, thus modulating immune and inflammatory responses in offspring [15]. In this study, CAE significantly increased serum levels of IgA (*p* = 0.002) and IgM (*p* = 0.001) in sows, although it did not significantly affect IgG levels (*p* = 0.449) (Figure 3E–G). These results suggest that supplementation with CAE during the peri-implantation phase can effectively alleviate systemic oxidative stress and inflammation, thus enhancing the overall health of sows.

### 3.3. Fecal Microbial Analysis

**Figure 4:**
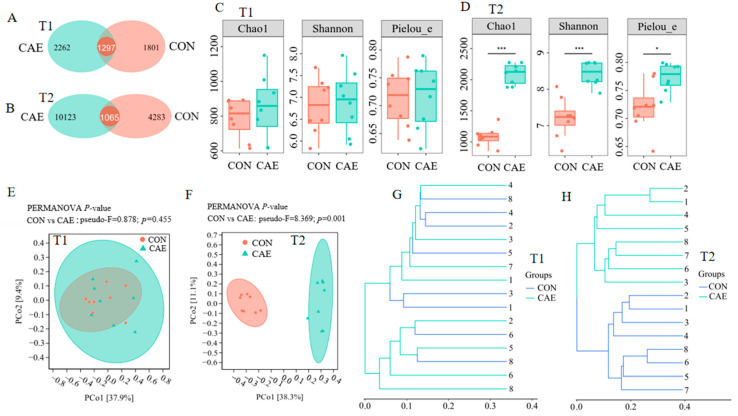
Effects of CAE supplementation during the peri-implantation phase on sow fecal microbial diversity. (**A**,**B**) The number of ASV that were copresent and separately detected in fecal samples, (**C**,**D**) fecal microbial alpha diversity (Chao1, Shannon, and Pielou_e indices), and beta diversity as shown by (**E**,**F**) principal coordinate analysis (PCoA) plots and (**G**,**H**) cluster analysis based on the Bray—Curtis distance (*n* = 8). T1 and T2 represent the stages 5 days before and 15 days into gestation, respectively. The term CON refers to the sows that were fed a standard basal diet without any supplementation, whereas CAE denotes the groups that were fed the basal diet supplemented with CAE at doses of 2000 mg/kg. * *p* < 0.05; *** *p* < 0.001.

To determine whether CAE supplementation affects the gut microbiota of sows during the peri-implantation phase, high-throughput sequencing of the 16S rRNA gene was conducted on fecal samples. Before the CAE treatment, fecal samples from sows in the CON and CAE groups had similar numbers of annotated ASV (CON: 3098 ASV; CAE: 3559 ASV). Compared with the CON group (5348), the CAE group presented a significantly greater number of annotated ASV (11,188) (Figure 4A,B). The alpha diversity indices (Chao1, Shannon, and Pielou’s) did not significantly differ (*p* = 0.404, *p* = 0.779, and *p* = 0.923) between the CON and CAE groups before CAE treatment but were significantly greater in the CAE group after treatment (*p <* 0.001, *p <* 0.001, and *p* = 0.011; Figure 4C,D). Beta diversity analysis of microbiota communities based on Bray—Curtis distances revealed no significant differences in microbiota community structure before CAE treatment (*p* = 0.455) but revealed significant alterations after CAE treatment (*p* = 0.001; Figure 4E–H).

**Figure 5:**
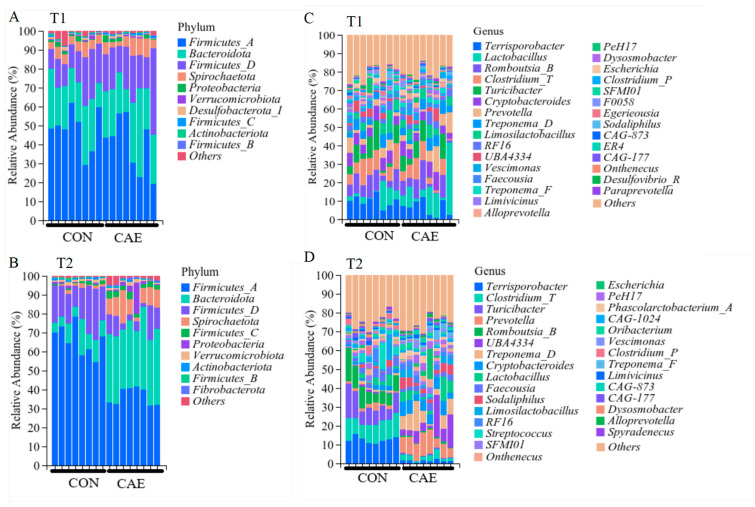
Effects of CAE supplementation during the peri-implantation phase on sow microbial communities at the phylum and genus levels. (**A**,**B**) The 10 most abundant microbial phyla identified in fecal samples and (**C**,**D**) the 30 most abundant microbial genera found in fecal samples (*n* = 8). T1 and T2 represent the stages 5 days before and 15 days into gestation, respectively. The term CON refers to the sows that were fed a standard basal diet without any supplementation, whereas CAE denotes the groups that were fed the basal diet supplemented with CAE at doses of 2000 mg/kg.

**Figure 6:**
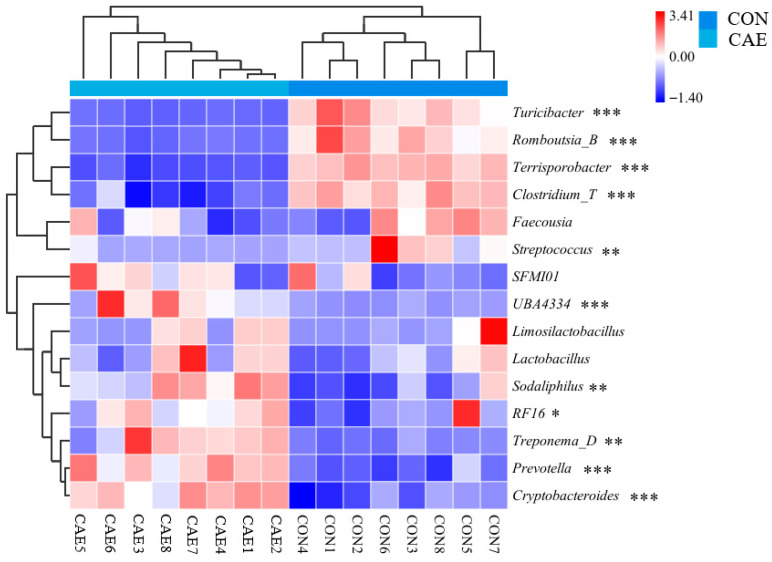
The impact of CAE supplementation during the peri-implantation phase on the 15 most prevalent microbial genera in sows, as illustrated by a heatmap (*n* = 8). The term CON refers to the sows that were fed a standard basal diet without any supplementation, whereas CAE denotes the groups that were fed the basal diet supplemented with CAE at doses of 2000 mg/kg. * *p* < 0.05; ** *p* < 0.01; *** *p* < 0.001.

Specifically, before CAE supplementation, the relative abundances of the major phyla were as follows: *Firmicutes_A* (CON: 48.22%, CAE: 40.27%), *Bacteroidota* (CON: 23.16%, CAE: 26%), and *Firmicutes_D* (CON: 17.38%, CAE: 22.91%) (Figure 5A). The 15 most abundant genera in the fecal microbiota of sows were *Terrisporobacter*, *Lactobacillus*, *Romboutsia_B*, *Clostridium_T*, *Turicibacter*, *Cryptobacteroides*, *Prevotella*, *Clostridium_D*, *Limosilactobacillus*, *RF16*, *Vescimonas*, *Faecousia*, *Treponema_F*, *Limivicinus*, and *Alloprevotella*; nevertheless, the abundance levels of these 15 genera did not significantly differ between the CON and CAE groups (Figure 5C). After CAE administration, the abundance of the major phyla *Firmicutes_A* (65.95%) and *Firmicutes_D* (18.72%) was altered in the CON group but that of *Firmicutes_A* (36.49%), *Bacteroidota* (35.72%), *Spirochaetota* (9.22%), *Firmicutes_D* (7.97%), and *Proteobacteria* (3.08%) was altered in the CAE group (Figure 5B). The 15 most abundant genera in the fecal microbiota of sows were *Terrisporobacter*, *Clostridium_T*, *Turicibacter*, *Prevotella*, *Romboutsia_B*, *UBA4334*, *Treponema_D*, *Cryptobacteroides*, *Lactobacillus*, *Faecousia*, *Sodaliphilus*, *Limosilactobacillus*, *RF16*, *Streptococcus*, and *SFM101* (Figure 5D). In the CON group, the dominant genera were *Turicibacter* (*p* < 0.001), *Romboutsia_B* (*p* < 0.001), *Terrisporobacter* (*p* < 0.001), *Clostridium_T* (*p* < 0.001), and *Streptococcus* (*p* = 0.002), whose relative abundances were significantly greater than those in the CAE group. In the CAE group, the dominant genera were *UBA4334* (*p* < 0.001), *Prevotella* (*p* < 0.001), *Treponema_D* (*p* = 0.001), *RF16* (*p* = 0.021), *Cryptobacteroides* (*p* < 0.001), and *Sodaliphilus* (*p* = 0.003), whose relative abundances were significantly greater than in the CON group (Figure 6). These results indicate that supplementation of the diet with CAE during the peri-implantation phase can significantly modify the fecal microbiota composition of sows.

### 3.4. Fecal SCFA Analysis

**Figure 7:**
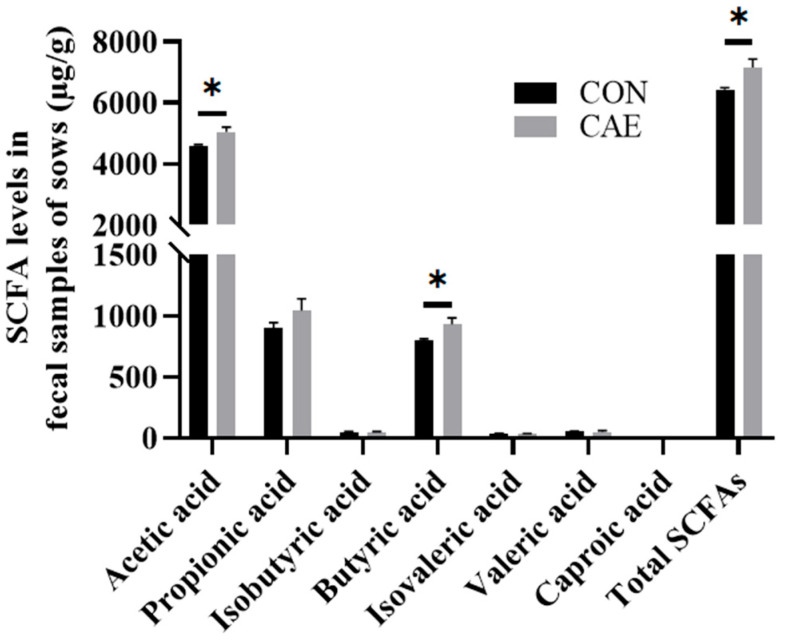
Effects of CAE supplementation during the peri-implantation phase on SCFA levels in sow fecal samples (*n* = 6). The term CON refers to the sows that were fed a standard basal diet without any supplementation, whereas CAE denotes the groups that were fed the basal diet supplemented with CAE at doses of 2000 mg/kg. * *p* < 0.05.

The gut microbiota primarily engages with the host via metabolites, which are key intermediates or products of microbial metabolism and play a crucial role in host endocrine regulation. The results indicate that compared with the CON group, the CAE group had markedly higher levels of acetic acid (*p* = 0.023), butyric acid (*p* = 0.037), and total SCFAs (*p* = 0.034) (Figure 7). Thus, the findings of this study suggest that alterations in the gut microbiota can modulate SCFA metabolism in sows.

### 3.5. Correlations Between the Microbiota and Antioxidant Indices and Between Microbiota and Reproductive Performance Indices

**Figure 8:**
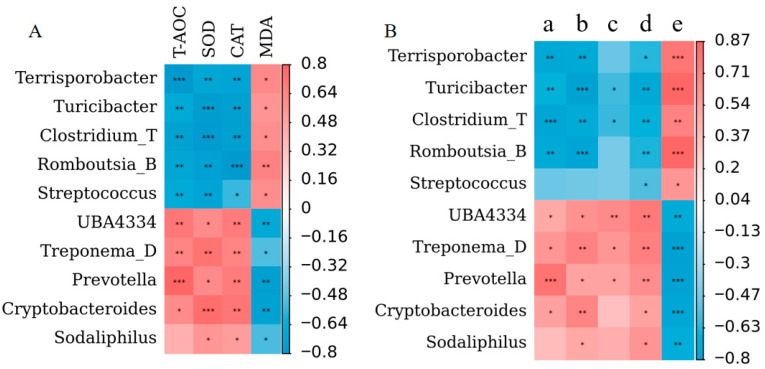
Heatmap of the Spearman correlation analysis. Correlation analysis was performed in two contexts: (**A**) between altered microbiota and antioxidant indices; (**B**) between altered microbiota and reproductive performance indices, including individual birth weight of live-born piglets (a), total litter birth weight of live-born piglets (b), litter size (c), total number of live-born piglets (d), and mortality rate (e) (*n* = 8). Red indicates a positive association, whereas blue indicates a negative association. * *p* < 0.05; ** *p* < 0.01; *** *p* < 0.001.

The abundances of *Terrisporobacter*, *Turicibacter*, *Clostridium_T*, *Romboutsia_B*, and *Streptococcus* were positively correlated with MDA but negatively correlated with T-AOC, SOD, and CAT. Conversely, *UBA4334*, *Treponema_D*, *Prevotella*, and *Cryptobacteroides* were negatively correlated with MDA and positively correlated with T-AOC, SOD, and CAT. Furthermore, *Sodaliphilus* was positively correlated with SOD and CAT but negatively correlated with MDA (Figure 8A). The data in Figure 8B indicate that *Clostridium_T* and *Turicibacter* were negatively correlated with the individual birth weight of live-born piglets, the total litter birth weight of live-born piglets, the litter size, and the total number of live-born piglets but positively correlated with the mortality rate. *UBA4334*, *Prevotella*, and *Treponema_D* exhibited opposite trends. *Terrisporobacter* and *Romboutsia_B* were inversely related to the individual birth weight of live-born piglets, the total litter birth weight of live-born piglets, and the total number of live-born piglets. *Streptococcus* was negatively correlated with the total number of live-born piglets. In contrast, these bacterial genera were positively correlated with the mortality rate. On the other hand, the abundances of *Cryptobacteroides* and *Sodaliphilus* were positively correlated with the total litter birth weight of live-born piglets and the total number of live-born piglets but negatively correlated with the mortality rate. *Cryptobacteroides* was also positively related to the individual birth weight of live-born piglets. These results suggest that *UBA4334*, *Treponema_D*, *Prevotella*, *Cryptobacteroides*, and *Sodaliphilus* may play important roles in reducing oxidative stress and improving reproductive performance in sows. More detailed data can be found in Table S1.

## 4. Discussion

Research has shown that the nutritional supply before embryo implantation and a sow’s health status are vital for the survival and development of the embryo [16]. During the initial phase of pregnancy, particularly before embryo implantation, sows often experience significant oxidative stress. This stress can obstruct embryonic development via DNA damage, apoptosis, and mitochondrial dysfunction [17,18]. Excessive oxidative stress can weaken a sow’s immune system, diminish antibody levels, and provoke chronic inflammation, all of which pose risks to the sow’s health and fetal development [17]. Moreover, the gut microbiota plays a key role in the female reproductive cycle, influencing reproductive performance at all stages [6]. In the present study, our results show that supplementation with CAE during the peri-implantation phase enhances serum antioxidant and immune indicators, optimizes the gut microbiota composition, and improves reproductive performance in sows, which supports our hypothesis.

*Eucommia* extract is a rich source of CGA. Extensive research has shown that CGA effectively inhibits the accumulation of ROS, mitigates oxidative stress, and reduces apoptosis in neurons and Sertoli cells [7,8]. Furthermore, CGA has been reported to significantly lower the levels of inflammatory factors in dairy cows [9]. Considering the advantageous attributes of CGA, CAE is likely to be highly effective at alleviating oxidative stress and inflammatory damage in sows during the peri-implantation period. In this study, sows were given two distinct doses of CAE (with a CGA content of 50%) during the peri-implantation period (from gestation day −5 to day 15). The outcomes were notably positive. Compared with the CON sows, the sows treated with CAE had a significantly greater total number of live-born piglets and higher birth weights of live-born piglets. CAE treatment also markedly increased serum antioxidant levels, as indicated by increased T-AOC, SOD, and CAT levels, along with a marked reduction in MDA levels. Furthermore, CAE administration led to substantial increases in serum IgA and IgM levels. IgM, the first immunoglobulin produced in the body, has strong bactericidal and immunomodulatory functions [19]. IgA, which makes up 10% to 20% of total serum immunoglobulins, is a crucial component of the mucosal defense system in animals and has antibacterial and antiviral properties [20]. Hence, CAEs play crucial roles in enhancing early embryonic development and improving sow reproductive performance.

The gut microbiota composition can affect host endocrine metabolism and reproductive performance. However, the impact of CAE on the gut microbiota of sows during peri-implantation is unclear. To investigate this, 16S rRNA analysis was conducted on pregnant sows. Initially, no notable differences in alpha or beta diversity were detected between the fecal samples of the CON and CAE groups. After CAE administration, the alpha diversity index in the CAE group increased significantly, and the beta diversity also significantly differed from that in the CON group. These findings indicate that CAE feeding altered the gut microbiota composition. Specifically, significant differences in the relative abundance of key genera, including *Terrisporobacter*, *Turicibacter*, *Clostridium_T*, *Romboutsia_B*, *Streptococcus*, *UBA4334*, *Treponema_D*, *Prevotella*, *Cryptobacteroides*, and *Sodaliphilus*, were observed between the two groups. Elevated levels of *Turicibacter* can disrupt bile acid levels, impair vitamin D absorption and metabolism, and reduce embryonic developmental capacity [21]. Furthermore, higher *Turicibacter* levels are linked to the accumulation of oxidative stress-related metabolites in the gut, which can enter the bloodstream and negatively affect embryonic development quality [21]. Similarly, *Romboutsia* has been shown to have adverse effects on gut health and is linked to increased levels of circulating inflammatory factors, such as IL-1β [22]. These inflammatory factors can travel through the bloodstream and negatively affect the development of embryos, thus reducing their potential for successful implantation [23]. *Terrisporobacter* is an anaerobic pathogen that has been shown to induce oxidative stress [24,25,26]. Moreover, *Streptococcus* is a major pathogen of swine endometritis and is associated with intestinal inflammation [27]. Although *Clostridium* is generally beneficial to host health under normal circumstances, certain strains may trigger an inflammatory response when the gut microbiome is imbalanced, leading to gut disease [28], which in turn affects host reproductive performance. The decrease in the abundance of *Turicibacter*, *Romboutsia_B*, *Terrisporobacter*, *Streptococcus*, and *Clostridium_T* in the CAE group was consistent with the increased antioxidant capacity and immune function observed in these sows. These findings may explain why the abundances of these bacteria were negatively correlated with reproductive performance and antioxidant indicators.

In contrast, *UBA4334* can increase the ability to metabolize specific nutrients [29]. Similarly, *Prevotella* and *Treponema* can utilize xylanases, mannanases, and β-glucanases to break down polysaccharides in grain cell walls, which in turn facilitates the fermentation of undigested carbohydrates into SCFAs [30,31]. Essential for gut health, SCFAs offer anti-inflammatory benefits and supply energy to the host [32] while also supporting embryo survival and early development [33]. This improved utilization of carbohydrates increases energy acquisition efficiency, which in turn provides greater energy support for embryonic development. Other genera, such as *Cryptobacteroides*, also contribute to carbohydrate breakdown and energy supply for the host [34]. Additionally, the newly discovered genus *Sodaliphilus* carries a variety of carbohydrate-active enzymes (CAZymes) that help break down complex carbohydrates, such as cellulose, arabinose, and mixed ligand glucans, into simple sugars, providing energy to the host [35]. Therefore, the increased presence of *UBA4334*, *Treponema_D*, *Prevotella*, *Cryptobacteroides*, *and Sodaliphilus* in the gut environment of sows may increase the absorption and utilization of nutrients in feed, indirectly supporting embryonic development. This aligns with the high reproductive performance observed in the CAE sow group.

Subsequent targeted metabolomics analysis of SCFAs revealed significantly higher levels of acetic acid, butyric acid, and total SCFAs in fecal samples from the CAE group than in those from the CON group. These elevated SCFA levels may further explain the increased abundance of SCFA-producing bacteria observed in the CAE sow group. Additionally, these findings not only further validate the potential benefits of CAE in enhancing antioxidant capacity, reducing inflammation, and supporting energy metabolic pathways but also highlight the regulatory role of CAE on the gut microbiota and its importance in reproductive health. Nevertheless, the underlying mechanisms have not been fully elucidated. Future research could examine the role of the gut microbiota in enhancing the reproductive performance of sows using approaches such as fecal microbiota transplantation. Moreover, further investigation into the role of the gut microbiota in SCFA biosynthesis is warranted. These strategies may offer deeper insights into the interplay between oxidative stress, the microbiome, and reproductive performance.

## 5. Conclusions

Dietary supplementation with CAE during the peri-implantation period may mitigate host oxidative stress, bolster maternal antioxidant and immune functions, and modulate the gut microbiota, thus enhancing reproductive performance in sows. These findings support our hypothesis and indicate that supplementation with CAE is beneficial for improving sow reproductive outcomes. This discovery provides an important scientific basis for future research and applications.

## Figures and Tables

**Table 1:** Basic dietary composition and nutritional levels for sows.

**Ingredient (%)**	**Content**	**Nutrient Levels ^§^**	**Content**
Corn	74.5	Crude protein (%)	12.50
Wheat bran	8.8	Calcium (%)	0.82
Soybean meal	12	Total phosphorus (%)	0.58
Soybean oil	1.2	Metabolizable Energy (MJ/kg)	12.62
Limestone	1.2		
Dicalcium phosphate	1.2		
Sodium chloride	0.3		
Choline chloride (50%)	0.1		
L-lysine	0.26		
DL-Methionine	0.24		
Vitamin premix ^†^	0.1		
Mineral premix ^‡^	0.1		
Total	100		

^†^ The vitamin supplement in the feed composition offered the following per kilogram: 5000 IU of vitamin A, 1000 IU of vitamin D3, 50 IU of vitamin E, 0.5 mg of vitamin K3, 5.0 mg of vitamin B1, 10.0 mg of vitamin B2, 6.0 mg of vitamin B6, 0.025 mg of vitamin B12, 10 mg of niacin, 20 mg of pantothenic acid, and 2.0 mg of folic acid. ^‡^ The mineral premix provides the following per kilogram of feed: copper, 20 mg; iron, 120 mg; zinc, 125 mg; manganese, 50 mg; selenium, 0.3 mg; and iodine, 0.5 mg. ^§^ Calculated value.

## Data Availability

The datasets generated during this study are available from the corresponding author upon reasonable request. The 16S rRNA sequencing data have been uploaded to the NCBI SRA database under the accession number PRJNA1287044.

## Supplementary Materials

The following supporting information can be downloaded at: https://www.mdpi.com/article/10.3390/vetsci12090857/s1, Table S1: The relationship between altered microbiota and both antioxidant and reproductive performance indicators.
